# Smoking status and changes in thyroid-stimulating hormone and free thyroxine levels during a decade of follow-up: The Tehran thyroid study 

**DOI:** 10.22088/cjim.11.1.47

**Published:** 2020

**Authors:** Hoda Kadkhodazadeh, Atieh Amouzegar, Ladan Mehran, Safoora Gharibzadeh, Fereidoun Azizi, Maryam Tohidi

**Affiliations:** 1 Endocrine Research Center, Research Institute for Endocrine Sciences, Shahid Beheshti University of Medical Sciences, Tehran, Iran; 2 Department of Epidemiology and Biostatistics, Research Center for Emerging and Reemerging Infectious Disease, Pasteur Institute of Iran, Tehran, Iran; 3 Prevention of Metabolic Disorders Research Center Research Institute for Endocrine Sciences, Shahid Beheshti University of Medical Sciences, Tehran, Iran

**Keywords:** Hypothyroidism, Hormones, Thyroid, Smoking

## Abstract

**Background:**

Smoking can cause thyroid disorders; the aim of the present study was to investigate the association between smoking status and changes in thyroid hormone levels among adult males during a decade long follow-up of in the Tehran Thyroid Study (TTS).

**Methods:**

Data of 895 adult males (smokers=115, non-smokers=691, ex-smokers=89) participants of the TTS without any previously known thyroid disease were analyzed. To examine trends of changes in thyroid hormone levels in these three groups, generalized estimating equation models were used. The interaction between the smoking status and each phase of the study was checked in a separate model.

**Results:**

Age and BMI adjusted trends of free thyroxine (FT4) demonstrated a non-significant decrease in participants (P=0.121) and thyroid-stimulating hormone (TSH) gained a significant average increase value over time in the total population (adjusted marginal mean of TSH=1.15 mU/L in phase 1, vs. 1.75 mU/L in phase 4, P<0.0001). Of the three groups, non-smokers and ex-smokers showed statistically significant increases in TSH during the follow-up period, whereas the smoker group had lower increases in TSH levels, changes from phase 1 until phase 2 among smokers were 38.46%, vs 43.54% and 52.94% in the ex and non-smokers, respectively.

**Conclusion:**

TSH was lower and FT4 was higher in smokers compared with the other smoker groups, although TSH level shows no decreasing trend over time in this group. The increasing trend of TSH in smokers was similar to ex and non-smokers. No difference was seen in FT4 trends among the smoking groups.

Scientific evidence shows that smoking is the most important and preventable risk factor for health problems and mortality (1). According to a WHO report, the total number of smokers is increasing in the whole world (2). Tobacco smoke contains about 4800 substances, including at least 200 toxicant and 80 known or suspected carcinogens (3). Effects of smoking on thyroid disorders have caused major concern, some  studies demonstrated that smoking did not have any significant relationship with thyroid functions (4), while others reported that smoking is associated with a decreased level of anti‐thyroid peroxidase antibodies (anti‐TPO Ab) and thyroid‐stimulating hormone (TSH) levels (5-7).TSH and free thyroxine (F4) levels are important indicators used to diagnose thyroid disorders (8) and assessing thyroid function according to smoking status. A population-based study showed lower mean of TSH values in smokers compared with non or former smokers (9).

Another study conducted in a Korean population reported mean serum TSH level in current smokers was significantly lower than that in non-smokers (10). Jorde et al. also revealed that TSH level (adjusted for age, sex) and BMI was lower in smokers compared to nonsmokers (11). 

**Objectives: **Although many studies have been conducted about the relationship between smoking and thyroid disorders, especially TSH, most theses are cross-sectional studies and few studies have assessed the changes of thyroid hormone levels in smoker populations over time in a cohort study. Considering the limited available data, the aim of the present study was to investigate the association between smoking status and changes in thyroid hormone levels among adult males during a decade long follow-up in the Tehran thyroid study (TTS).

## Methods

**Study population: **Detailed descriptions of TTS have been published elsewhere (12, 13)briefly, the TTS, a population-based cohort study performed on a representative sample of residents of district No.13 of Tehran (the capital city of Iran), was designed to evaluate the prevalence and incidence of thyroid diseases and related risk factors in adult population. TTS has two major components: a cross-sectional study (1999-2001) and a prospective follow-up study at intervals of approximately 3 years.

**Fig. 1:**
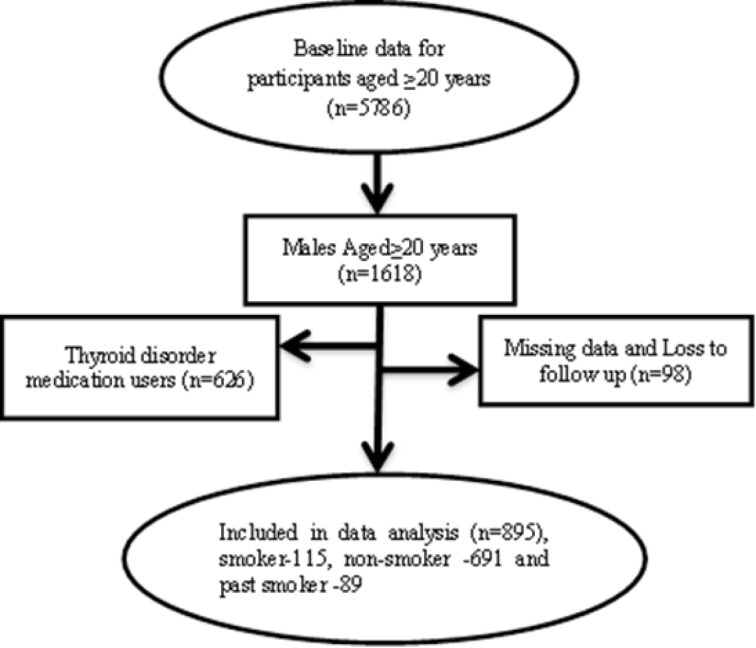
Participant’s selection in the study

In the current study, from a total 4174 participants with aged ≥20 years, we excluded women for low prevalence of smoking (n=1968), those using any medications related to thyroid disorders (n=786), those with any thyroid dysfunction or thyroid surgery history or cancer (n=786) and participants who had no follow up or missing data (n=321). Eventually 895 subjects with complete data, from 1999 until March 2012 were enrolled in the present study (figure 1). The median follow-up duration was 9.57±0.87 years. The study design was approved by the Institutional Review Board (IRB) of the Research Institute for Endocrine Sciences (RIES), Shahid Beheshti University of Medical Sciences.

**Clinical and laboratory measurements: **All participants, after signing informed written consent forms were invited to participate in the TTS. Baseline and follow up information including age, smoking status, medication use and thyroid disorders history were collected by trained interviewers, using a standard questionnaire. Details of anthropometric measures obtained using standard protocols, including weight and height have been reported elsewhere (14). Body mass index (BMI) was calculated by dividing weight (kg) by square of height (m^2^). A venous blood sample was drawn between 7:00 and 9:00 AM after 12–14 hours of overnight fasting for each participant. Free thyroxine (FT4) and thyroid stimulating hormone (TSH) were measured on −70°C stored serum samples by electrochemiluminescence immunoassay using Roche Diagnostics kits & Roche/ Hitachi Cobas e-411 analyzer (GmbH, Mannheim, Germany). The sensitivity of TSH and FT4 measurements were 0.005 μIU/ml and 0.30 pmol/l, respectively. Lyophilized quality control material (Lyphochek Immunoassay plus Control, Bio-Rad Laboratories) was used for accuracy assessment of hormonal assays; intro- and inter-assay coefficients of variation for TSH were 1.4% and 4.6% and for FT4 assay were 1.3 % and 3.3 %, respectively. Details of laboratory assessments have been reported elsewhere (14). 

**Definition of terms: **Current smokers were defined as participants who were smoking cigarettes daily or occasionally, as well as those who used water pipe or pipe at the time of the study. Ex-smokers were defined as participants who used to smoke in the past. All participants who had never smoked were called non-smokers. 

**Statistical analysis: **Baseline characteristics of participants are reported as mean±standard deviation (SD) for continuous variables. TSH is reported as median and inter-quartile range (IQR); categorical variables are given as a number (percent). Normality assumption was checked using the Shapiro- Wilk test. One-way ANOVA test (Kruskal-Wallis H test) was used to explore differences in descriptive baseline characteristics between the smoker, non-smoker and ex-smoker groups.

To analyze the effect of smoking status on the trend of TFT, we used generalized estimating equations (GEE), a statistical method that facilitates the analysis of longitudinal data for many different distributions. We used an exchangeable correlation structure with a random intercept for this study. At first step, models were developed at two hierarchical levels; age-adjusted (adjusting for age and phase) and multivariate-adjusted (adjusting for age, examination phase, BMI, and smoking status) in the total population. In the second step, we developed the models in three groups, separately. Again, models were developed at two hierarchical levels; age-adjusted (adjusting for age and phase) and multivariate-adjusted (adjusting for age, examination phase, and BMI). P<0.05 were considered significant. All analyses were performed using STATA statistical software (StataCorp. 2013. *Stata Statistical Software: Release 13*. College Station, TX: StataCorp LP)

## Results

Overall, 895 men participants, with a mean age of 41.67 (SD=14.15) years were included in the analysis; of these, 115 (12.85%) cases were current smokers, 89 (9.94%) were ex-smokers, and approximately 691(77.21%) of them had never smoked previously. As shown in table 1, no significant difference was observed in mean BMI among the three groups. Mean TSH among the non-smoker, ex-smoker and smoker groups were 1.35, 1.35 and 0.97, respectively (P=0.01), similar to the means for FT4 was 1.23, 1.24 and 1.27 respectively (P=0.047).

We observed a significant decrease in FT4 levels during the study; however, that trend did not remain significant after adjusting for BMI and smoking status (P=0.12, table 2). TSH levels significantly increased during the study in the total population of both models. (p<0.01, table 2).

Table 3 shows the results of GEE for the three groups. TSH increased significantly over a decade in three smokers (mean TSH= 0.93 and 1.26 in visit 1 vs. visit 4), ex-smoker (mean TSH= 1.24 and 1.78 in visit 1 vs. visit 4) and non-smoker group (mean TSH=1.19 and 1.82 in visit 1 vs. visit 4). We observed a statistically significant decrease in FT4 levels during the study only in non- smokers (p<0.01).

**Table 1:** Baseline characteristics of study population by smoking status: Tehran thyroid study, 1999–2012

	**Total **	**Non-smoker**	**Ex-smoker**	**Smoker**	**P-value**
	**(n=895)**	**n=691**	**n=89**	**N=115**	
Age (years)*	41.67±14.15	40.14±8.42	52.12± 0.11	38.10± 8.14	<0.01
BMI (kg/m2)	25.83±4.05	25.40±9.11	26.33±2.94	25.32±2.94	<0.183
TSH (mU/L)**	1.31(0.79, 1.93)	1.35(0.84, 1.98)	1.35(0.77, 1.92)	0.97(0.59, 1.49)	<0.01
FT4 (ng/dl)	1.27±0.21				0.047
TPOAb (IU/ml)	5.2(3.09, 9.13)	5.4(3.25, 9.42)	5.28(3.04, 8.17)	3.81(2.44, 8.05)	0.002

*Data presented as Mean ±SD, ** Median (IQR)

TSH; Thyroid Stimulating Hormone, FT4; Free Thyroxine

**Table 2:** Age-adjusted and multivariate adjusted mean levels* of TSH, FT4 by phases

	**Phase1** **(1999-2001)**	**Phase 2** **(2002-2005)**	**Phase 3** **(2005-2008)**	**Phase 4** **(2008-2011)**	**P-value for time trend**
Model 1*					
TSH (mU/L)	1.14(1.08, 1.22)	1.32(1.24, 1.40)	1.44(1.36, 1.53)	1.75(1.64, 1.86)	<0.0001
FT4 (ng/dl)	1.25(1.24, 1.26)	1.24(1.22, 1.25)	1.23(1.21, 1.25)	1.22(1.20, 1.23)	0.02
Model2**					
TSH (mU/L)	1.15(1.08, 1.22)	1.32(1.24, 1.40)	1.44(1.36, 1.53)	1.75(1.64, 1.86)	<0.0001
FT4 (ng/dl)	1.24(1.23, 1.26)	1.24(1.22, 1.25)	1.23(1.22, 1.24)	1.22(1.21, 1.23)	0.121

*Adjusted for age and phases. **Values are adjusted for age, phases, BMI and smoking status.

**Table 3:** The results of generalized estimating equations (GEE) for the three groups

	**Phase1** **(1999-2001)**			**Phase 2** **(2002-2005)**			**Phase 3** **(2005-2008)**			**Phase 4** **(2008-2011)**		
	Smoker	Ex-Smoker	Non-smoker	Smoker	Ex-Smoker	Non-smoker	Smoker	Ex-Smoker	Non-smoker	Smoker	Ex-Smoker	Non-smoker
Model 1*												
TSH	0.90 (0.77-1.05)	1.25 (0.99-1.57)	1.18 (1.10-1.26)	1.09 (0.95-1.26)	1.26 (1.01-1.56)	1.36 (1.27-1.46)	1.14 (0.87-1.31)	1.33 (1.07-1.64)	1.52 (1.42-1.63)	1.37 (1.17-1.59)	1.77 (1.41-2.22)	1.82 (1.70-1.95)
FT4	1.27 (1.24-1.30)	1.21 (1.16-1.25)	1.25 (1.24-1.27)	1.27 (1.24-1.30)	1.19 (1.15-1.23)	1.24 (1.22-1.25)	1.26 (1.23-1.29)	1.22 (1.18-1.26)	1.22 (1.20-1.24)	1.27 (1.23-1.30)	1.21 (1.17-1.25)	1.21 (1.19-1.22)
Model2**												
TSH	0.91(0.77-1.06)	1.25 (1.00-1.58)	1.19 (1.11-1.27)	1.09 (0.95-1.27)	1.28 (1.03-1.59)	1.37 (1.27-1.47)	1.13 (0.98-1.31)	1.32 (1.07-1.64)	1.52 (1.42-1.63)	1.36 (1.16-1.59)	1.77 (1.41-2.23)	1.82 (1.70-1.95)
FT4	1.27 (1.23-1.30)	1.20 (1.16-1.24)	1.24 (1.23-1.26)	1.27 (1.23-1.30)	1.19 (1.15-1.23)	1.24 (1.22-1.25)	1.26 (1.23-1.29)	1.22 (1.18-1.26)	1.22 (1.21-1.24)	1.28 (1.24-1.31)	1.21 (1.17-1.25)	1.21 (1.19-1.22)

Values are adjusted means (95% confidence intervals) from generalized estimating equations to account for correlated observations. *Adjusted for age and phase. **Values are adjusted for age, phase, BMI.

## Discussion

To the best of our knowledge, the present study is the first study conducted within large cohort study in the Middle East region reporting the trend of thyroid hormones based on their smoking status among adult men. Results showed that FT4 levels remained more stable in individuals with different smoking status, compared to TSH, which showed dramatic increases in all three groups. Smokers however, generally experienced lower rates of change in their TSH, compared to the other groups. Results consistent with findings of other studies demonstrating that smoking is associated with lower TSH concentrations. 

Regarding cross-sectional analysis, we found that smokers had lower serum TSH levels than the two groups. Previous studies show similar results, i.e. smoking is associated with lower serum TSH level (9, 10, 15). One study conducted in an iodide replete-area showed that smoking reduced the risk of subclinical hypothyroidism in people with high iodine intake. Results of this study suggest that iodine intake is an important variable to elucidate associations between smoking and TSH(15). Although, we observed a significant increasing trend in TSH among the three smoking groups, the mean percentage change in TSH levels between these three groups differs. Previous studies show that, in former smokers, TSH increases with time after smoking cessation; it is only 18 years in men and 5 – 10 years in women that these groups achieve the never smoker status (16-18). However our study shows different results, the magnitude of the increased mean value of TSH in the smoking group was lower than the ex and non-smoker groups after 10 years (38.46 vs 43.54 and 39.56 respectively), Findings not in agreement with others groups. These results suggest that TSH level did not show decrease over time in the smoker group and trend of increasing in TSH level is similar to that of non-smoker population. It is difficult to compare the results of the present study with data of other epidemiologic studies on smoking and thyroid abnormalities as different methods are used for different aims. These cross-sectional findings provided some insight on the lower levels of TSH among smokers vs non-smokers, further follow up studies are needed to determine and confirm trends of TSH changes in this group.

The result of the present study revealed that mean serum FT4 concentrations in smokers are lower than non-smokers, findings in line with thyroid hormone changes within normal pituitary feedback; however, FT4 trend did not differ over 10 years. Data show conflicting results regarding the associations between FT4 concentrations and smoking; some studies reveal lower FT4 levels among smokers vs nonsmokers (16, 19). Findings contrary to those of two Korean studies showing that FT4 concentrations did not differ based on smoking status (7, 15). The present study has several limitations, we classified smoking status based on self-reports and the possibility of misclassification cannot be neglected. Another limitation is that we have not considered different categories of smoking for establishing the dose-response effect of smoking status. As for the strengths of the present study, we have used the data of an ongoing cohort study with a relatively long period of follow-up to assess the trends of thyroid hormones overtime. 

In conclusion, the results show that although mean TSH values among smokers are much lower than non-smokers and ex-smokers at baseline (cross-sectional comparison), the 10-year-trend showed a 38.46 increase from baseline values. Results suggest the TSH level showed no decreasing trend over time among smokers. For TSH the increasing trend in smokers was similar to those of ex and non-smokers, despite previous insight on lower TSH values in smokers, although the increasing trend was lower in smokers. FT4 was higher in smokers at baseline but showed no difference between the smoking groups. Furthermore, follow-up studies are needed to determine trends of TSH change in this group.
